# Improved detection of spinal cord lesions using an axial T2-weighted TSE sequence with full spinal cord coverage compared to sagittal T2-weighted TSE and STIR sequences in multiple sclerosis: a prospective study

**DOI:** 10.1007/s00234-025-03813-9

**Published:** 2025-10-21

**Authors:** Christian Thaler, Vincent Geest, Lukas Meyer, Charlotte Schubert, Christoph Heesen, Jens Fiehler, Susanne Gellißen

**Affiliations:** 1 Department of Neuroradiology, University Medical Center Hamburg-Eppendorf, Hamburg, Germany; 2 Department of Neurology, University Medical Center Hamburg-Eppendorf, Hamburg, Germany

**Keywords:** Multipe sclerosis, Spinal cord, Lesion, Magnetic resonance imaging, T2-weighted sequences

## Abstract

**Objective:**

This study aimed to compare the detection rates and inter-rater agreements of the sagittal T2w-TSE and sagittal short tau inversion recovery (STIR) sequence versus the axial T2w-TSE sequence with full spinal cord coverage in identifying spinal cord lesions in patients with suspected demyelinating diseases and diagnosed multiple sclerosis (MS).

**Methods:**

104 patients were prospectively enrolled in this study and underwent MRI, including a sagittal T2w-TSE and STIR sequence, as well as an axial T2w-TSE sequence with full spinal cord coverage. Two experienced neuroradiologists, blinded to clinical parameters, independently evaluated the scans in separate sessions. After blinded readings, raters re-evaluated all sequences to assess if lesions could be retrospectively identified in other sequences.

**Results:**

Spinal cord lesions were found in 81 patients. The highest inter-rater reliability was observed for the sagittal T2w-TSE sequence (κ = 0.73, 95%-CI 0.66–0.79), followed by the axial T2w-TSE (κ = 0.71, 95%-CI 0.63–0.79) and the sagittal STIR sequence (κ = 0.65, 95%-CI 0.58–0.73). The axial T2w-TSE sequence demonstrated superior lesion detection rates, identifying significantly more lesions (*n* = 361) compared to the STIR (*n* = 293) and T2w-TSE sagittal (*n* = 224) sequence (*p* < 0.001).

**Conclusion:**

Axial T2w-TSE sequences with full spinal cord coverage provide superior lesion detection compared to sagittal sequences and should be included in standard MRI protocols for MS patients. They may accelerate meeting MRI criteria for MS, improve monitoring of disease progression, and enhance prediction of future disability.

## Introduction

 Multiple sclerosis (MS) is a chronic neuroinflammatory disease of the central nervous system, affecting over 2.8 million people worldwide [1]. To diagnose MS and evaluate its disease progression, magnetic resonance imaging (MRI) is an essential tool to detect inflammatory lesions in the central nervous system (CNS) disseminated in time and space. For the dissemination in space spinal cord lesions were incorporated into the McDonald Diagnostic Criteria for MS in 2005 and have since remained a key component until the latest version (2017 revised McDonald criteria) [2]. This is comprehensible given that up to 85% of all patients with clinically diagnosed MS reveal spinal cord lesions on MR imaging [3, 4]. Spinal cord lesions are not only important to fulfill the diagnostic criteria but have also been recognized as a risk factor for quicker progression to definite MS in patients with clinical isolated syndrome (CIS) and as a stronger predictor of future disability compared to brain MRI measures [5–7]. Hence, it is of utmost importance to reliably detect spinal lesions in patients with suspected demyelinating diseases.

In a 2021 published consensus statement by the Magnetic Resonance Imaging in Multiple Sclerosis (MAGNIMS), Consortium of Multiple Sclerosis Centres (CMSC), and North American Imaging in Multiple Sclerosis Cooperative (NAIMS) on the use of MRI in MS patients, a standardized protocol was recommended for the detection of spinal cord lesions [8]. This protocol should include at least two of the following three sagittal sequences: T2-weighted (T2w) spin echo with moderately long echo times, proton density-weighted echo, or short tau inversion recovery (STIR). An axial acquired T2w sequence was only considered optional. In the updated recommendations from 2024 an axial T2w sequence with coverage of the cervical spinal cord has been added [9]. This is a point of critical debate since axial T2w sequences with full spinal cord coverage have shown superior detection rates compared with sagittal scans but are also supposed to be time consuming [4, 10]. 

The aim of this study was to compare the detection rates and inter-rater agreement between sagittal and axial acquired scans in the detection of spinal cord lesions in suspected demyelinating disease or disease monitoring of MS patients. We hypothesized that axial T2w sequences with full spinal cord coverage have higher lesion detections rate than sagittal acquired scans with comparable inter-rater reliability.

## Methods

The study was approved by the local Ethical Committee following the guidelines of the Declaration of Helsinki and patients provided written informed consent. 104 patients were consecutively enrolled in this prospective study between December 2022 to March 2023. All patients were referred to our department from the MS day hospital and received an MRI of the spinal cord. Inclusion criteria were as follows: age 18–70 years; diagnosed MS according to the 2017 revised McDonald criteria [3], clinical symptoms or brain imaging findings suspicious of a demyelinating disease; absence of neurologic conditions other than a chronic demyelinating disease. Detailed information about our study population is given in Table 1.

**Table 1:** Patients’ characteristics. For age and disease duration the mean and standard deviation, for EDSS the median and interquartile range is displayed. *Miscellaneous* including patients with clinical or imaging findings suspicious of chronic demyelinating disease, such as clinically/radiologically isolated syndrome (CIS/RIS), transverse myelitis or optic neuritis. *RRMS = relapsing remitting multiple sclerosis*,* SPMS = secondary progressive multiple sclerosis*,* PPMS = primary progressive multiple sclerosis*,* NMOSD = neuromyelitis Optica spectrum disorder*

Sex	77 female, 24 male
Age	35.7 (± 10.4) years
Diagnosis	54 RRMS, 3 SPMS, 13 PPMS, 3 NMOSD, 28 Miscellaneous
Disease/Symptom duration	5.8 (± 7.2) years
EDSS	2 (1–3)

### MR imaging protocol

All scans were performed on a 3 Tesla MR scanner (Magnetom Skyra, Siemens Medical Systems, Erlangen, Germany) and the MRI protocol included a sagittal T2w turbo spin-echo sequence (TSE) (Repetition Time (TR) = 3800 ms; Time of Echo (TE) = 95 ms; pixel size = 0.781 × 0.781 mm; Field of View (FOV) = 300 × 300 mm; slice thickness = 3 mm; no section gap; acquisition time = 3 min and 56 s), a sagittal short tau inversion recovery sequence (STIR) (TR = 3000 ms; TE = 48 ms; Inversion Time (TI) = 220 ms; pixel size = 0.781 × 0.781 mm; FOV = 300 × 300 mm; slice thickness = 3 mm; no section gap; acquisition time = 5 min and 36 s) and a axial T2w-TSE (TR = 6240 ms; TE = 111 ms; pixel size = 0.625 × 0.625 mm; FOV = 200 × 200 mm; slice thickness = 3.5 mm; 50 sections without a section gap; acquisition time = 5 min and 15 s for 2 slabs or 6 min and 46 s for 3 slabs with full spinal cord coverage (including the tip of the odontoid process and the conus medullaris)).

### Lesion Detection

Lesion detection was performed by two board certified neuroradiologists with respectively 18 and 10 years of experience in MS imaging diagnostics and evaluation. Image evaluation was performed on a medical workstation on calibrated, high-resolution monitors.

The raters evaluated each sequence separately, blinded to the patients’ characteristics and rating results of the other sequences. In a first reading, the sagittal T2w-TSE images were presented to the raters and MS lesions were detected and marked by each rater. In a second reading, the sagittal STIR images and in a third reading the axial T2w-TSE images were presented to the raters. There was a two-week gap between each reading. The raters were unaware of the patient’s identity and all clinical and imaging details, particularly the results and images from the other corresponding scans. After completion of the blinded readings, the images were re-evaluated by each rater including all sequences and results of the previous blinded reading to assess whether the lesions could be identified retrospectively in the other sequences.

The location in the axial plane (midline or lateral), and position (cervical or thoracic), as well as craniocaudal extension and axial diameter were documented for each lesion.

### Statistical analysis

IBM SPSS 27.0. (IBM Corp., Armonk, NY, USA) was used for statistical analysis. Cohens’s Kappa was calculated to determine the inter-rater agreement and magnitude guidelines were applied according to Landis and Koch [11]. 

For further analysis, only lesions identified by both raters were included. Descriptive statistics are presented as frequencies for categorical variables and compared with Fisher’s Exact test, mean (standard deviation [SD]) for continuous normally distributed variables and compared with the unpaired t-test.

## Results

### Patient demographics

104 patients were enrolled in this study. Three patients had to be excluded due to excessive motions artifacts. No spinal lesions were detected by any rater in 20 of these patients and 81 patients had at least one lesion detected by either of the raters. Patients’ characteristics are displayed in Table 1.

### Inter-rater Reliability

Overall, Rater 1 (CT) detected 433 lesions and Rater 2 (SG) detected 446 lesions. The detection rates of each rater and for each sequence are displayed in Table 2. Lesion detection in the sagittal T2w-TSE images offered the highest inter-rater reliability (κ = 0.73, *p* < 0.001), followed by the axial T2w-TSE (κ = 0.71, *p* < 0.001) and lastly the sagittal STIR images (κ = 0.65, *p* < 0.001). A substantial agreement was found for lesions, that were detected in at least two sequences by each rater (κ = 0.76, *p* < 0.001). Also, we found a substantial agreement for lesions that were detected in the sagittal as well as in the axial T2w-TSE (κ = 0.69, *p* < 0.001) by each rater, but only a moderate agreement for lesion that were detected in the sagittal T2w-TSE as well as in the sagittal STIR images (κ = 0.61, *p* < 0.001).

**Table 2:** Number of detected lesions by each rater in each sequence after the first blinded reading and corresponding κ values indicating the inter-rater agreement

	R1	R2	κ (95%-CI)
T2w-TSE sag	262	253	0.73 (0.66–0.79)
STIR sag	325	338	0.65 (0.58–0.73)
T2w-TSE ax	401	388	0.71 (0.63–0.79)
Lesion in any 2 sequences	319	318	0.76 (0.70–0.82)
Lesions in T2w-TSE sag and STIR	244	307	0.61 (0.54–0.68)
Lesions in T2w-TSE sag and ax	248	227	0.69 (0.63–0.76)
Total lesions detected	433	446	n/a

### Lesion evaluation

**Fig. 1:**
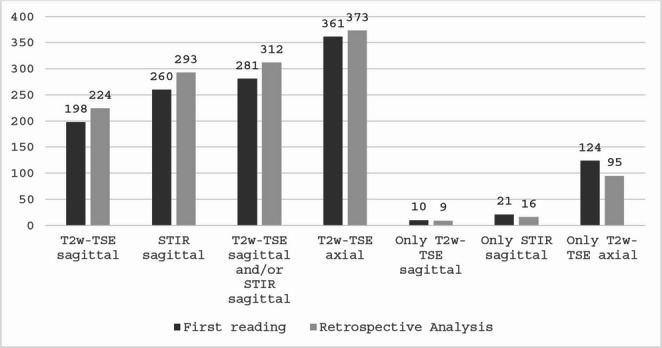
Graph displaying the total number of lesions detected in the corresponding sequence (sagittal T2w-TSE, STIR, and axial T2w-TSE), and the lesion number marked on the corresponding scans only without corresponding correlates in the other plane

**Fig. 2:**
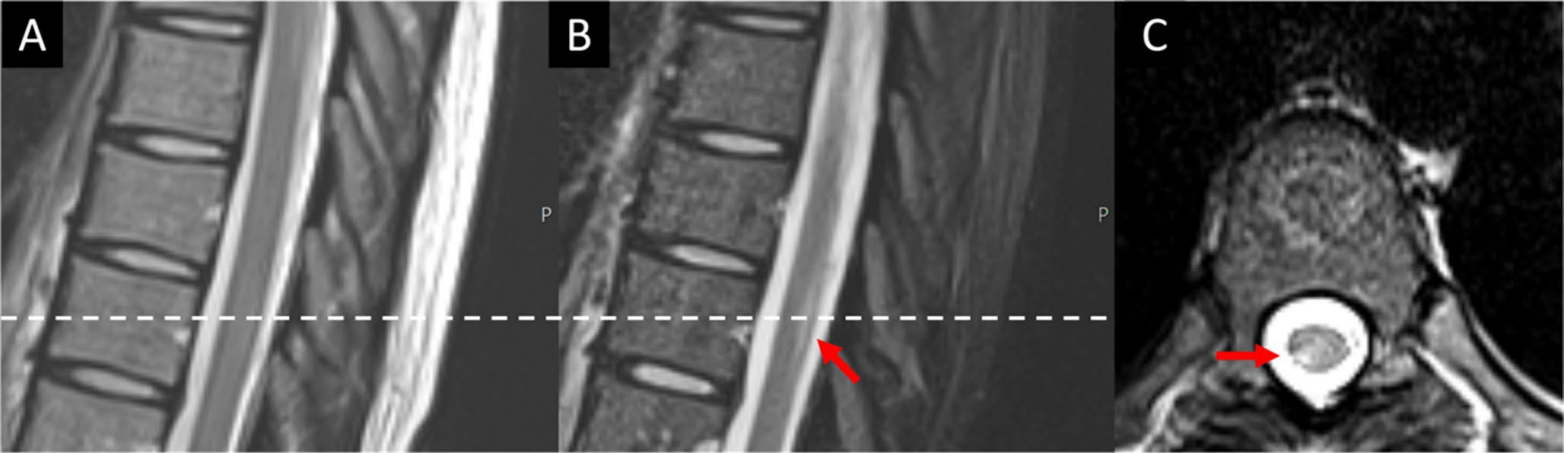
Example of a lesion that was detected by both raters on the axial T2w-TSE images (**C**) and only retrospectively on the STIR images (**B**). The lesion could not be identified on the sagittal T2-TSE images (**A**). Of note, there was slight patient movement between the acquisition of the sagittal T2w-TSE and STIR sequence, explaining the slightly deviating depiction of the spinal cord. The white dotted line implicates the level of the axial image

For further analysis, only lesions that were detected by both raters, regardless of the sequence, were included. Overall, 408 lesion were detected by both raters in 73 patients (73/81). The median number of lesions identified per person was 5 (IQR 2–7). 220 lesions were detected in the cervical and 188 in the thoracic spinal cord. In the first reading, a significantly higher number of lesions were detected in the axial T2w-TSE images (*n* = 361) than in the STIR (*n* = 293, *p* < 0.001) and sagittal T2w-TSE images (*n* = 224, *p* < 0.001). In a second reading with all sequences available for the raters, 12 lesions could be retrospectively detected in the axial T2w-TSE, 26 lesions in the sagittal T2w-TSE and 33 lesions in the sagittal STIR images (see Fig. 1). After including retrospectively detected lesions, the detection rates of axial T2w-TSE images were still significantly higher than those of the other two individual sequences (both *p* < 0.001) but also compared to the combined detection rate of the T2w-TSE sagittal and STIR sequence (*p* < 0.001). Even after including only lesions that were at least 3 mm in their long axis, detection rates of axial T2w-TSE images (*n* = 329) were still significant higher compared to sagittal STIR (*n* = 273) and T2w-TSE (*n* = 212) images (both *p* < 0.001) (Fig. 2).

**Fig. 3:**
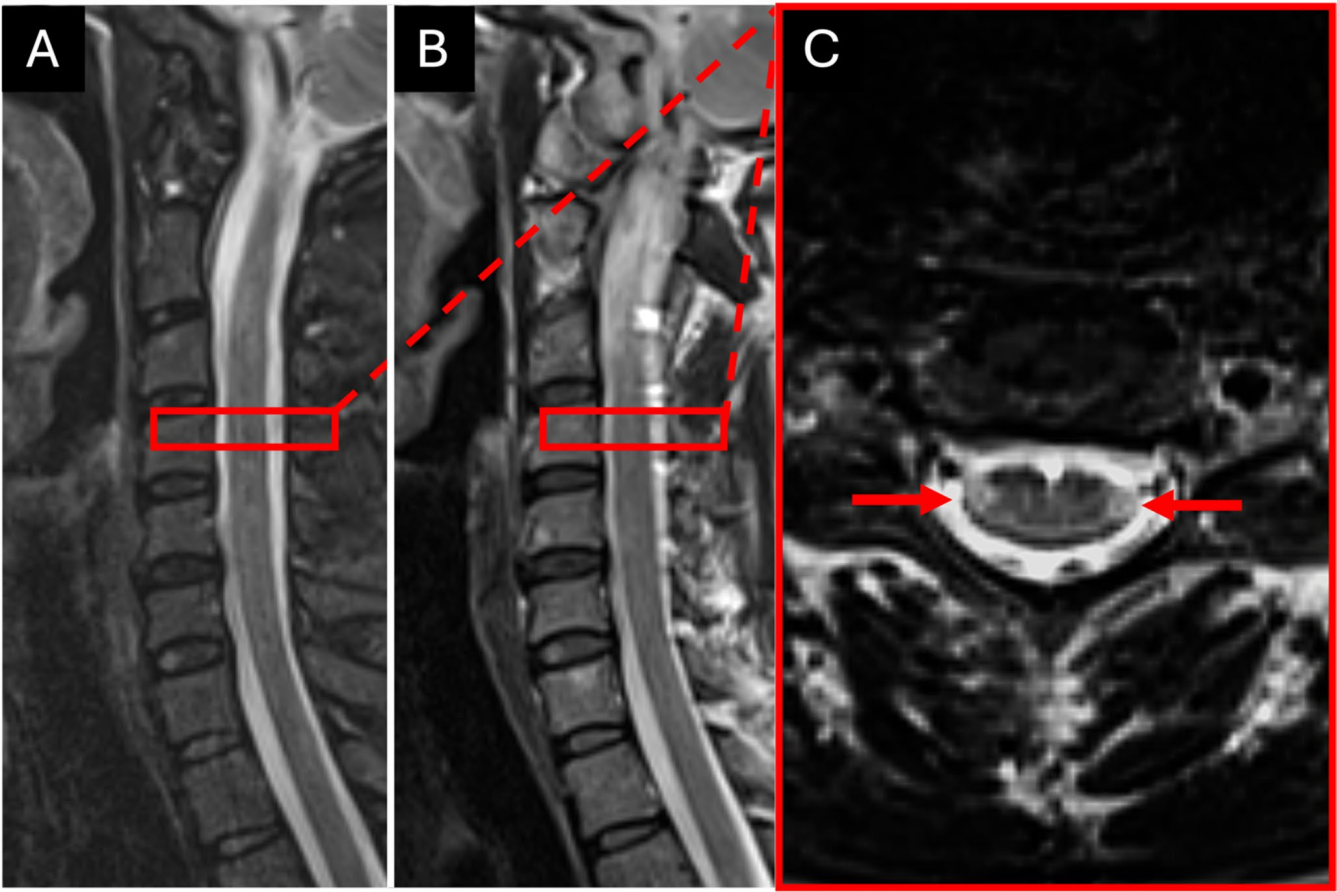
Example of two smaller spinal cord lesions (red arrows) with an axial diameter of 2 mm each and located laterally in the cervical spinal cord, that were not detected in the sagittal T2w-TSE (**A**) or STIR (**B**) but only in the axial T2w-TSE (**C**) images

From the 124 lesions that were only detected in the T2w-TSE axial images in the first reading, 29 lesions could be retrospectively detected in either the sagittal T2w-TSE or STIR images. In contrast, from the 21 lesions that were only detected in the STIR images in the first reading, 5 lesions could be retrospectively detected in either the sagittal or axial T2w-TSE images. Figure 3 gives an example of a retrospectively detected lesion.

After retrospective analysis 43 patients showed lesions that were detected in the axial T2w-TSE images but no other sequence. Two of these patients displayed only lesions visible on axial images and no lesions were detected on sagittal scans.

### Lesion characteristics

After retrospective analysis of all sequences, 95 lesions remained that were only seen by both raters in the axial T2w-TSE images but no other sequence. An example is given in Fig. 3. Compared to lesions that were also detected in the sagittal T2w-TSE or STIR images, these lesions were significantly smaller in their axial diameter and craniocaudal extension and were located more laterally in the spinal cord (see Table 3).

**Table 3:** Characteristics of lesions that were only detected in the axial T2w-TSE images compared to lesions that were also detected inn either the sagittal T2w-TSE or STIR images. **Boldface type** indicates statistically significant values

	Only detected in axial T2w-TSE (*n* = 95)	Also seen in sagittal T2w-TSE or STIR (*n* = 313)	*p* value
Axial diameter (mm)	3.26 (1.03)	4.16 (1.37)	**< 0.001**
Craniocaudal extension (mm)	6.78 (3.1)	9.48 (4.13)	**< 0.001**
Location in axial plane	46 (48.4%) lateral, 49 (51.6%) central	114 (36.4%) lateral, 199 (63.6%) central	**0.03**
Position (cervical vs. thoracic)	46 (48.4%) cervical, 49 (51.6%) thoracic	174 (55.6%) cervical, 139 (44.4%) thoracic	0.13

## Discussion

In contrast to recent recommendations [8, 9], we achieved highest detection rates for spinal cord lesions using an axial T2w-TSE sequence with full spinal cord coverage, followed by a sagittal STIR, and lastly a sagittal T2w-TSE sequence. This is not surprising, since former studies have already demonstrated the superior detection rates of axial T2w sequences with full or at least long spinal cord coverage compared to sagittal T2w sequences [10, 12]. However, these studies did not compare the detection rates of the axial acquisitions to sagittal STIR sequences. To our knowledge, this is the first study demonstrating that the axial T2w-TSE sequence detects more spinal cord lesions in MS patients than the STIR sequence, even when combined with the sagittal T2w-TSE sequence. Nevertheless, the STIR sequence does offer additional diagnostic value since it outperformed the sagittal T2w-TSE sequence in our study, which is in line with previously published results [13–15]. With the sagittal STIR sequence we detected 31% more lesions compared to the sagittal T2w sequence, which is similar to the results of Dietemann et al. or Nayak et al. who reported an increase of 46% and 35%. However, in the axial T2w-TSE images 67% more lesions could be detected than in the sagittal T2w-TSE images and 27% more lesions compared to the sagittal STIR sequence. Previous studies have also reported a significant increase in lesion detection using an axial T2w-TSE sequence compared to a sagittal T2w-TSE sequence, with 33.2 to 153.7% more lesions detected [10, 12]. So far to our knowledge, there are no previous studies comparing the detection rates between the sagittal STIR sequence and the axial T2w-TSE sequence.

Ninety-five lesions were only seen on axial images and could not be detected, even retrospectively, on the sagittal images. These lesions had a smaller diameter, a smaller craniocaudal extension and were more often located in the lateral spinal cord. Reasons for missing these lesions on sagittal images could be the recommended slice thickness of 3 mm of the sagittal sequences [8]. With a tapering transverse diameter of the spinal cord, which ranges in average between 13.2 mm at its widest level of C5 and 8.3 mm at T8 [16], it is possible that the spinal cord might only be fully captured in one slice and only displayed partially or with significant partial volume effects in the more lateral slices. Accordingly, more lesions were solely detected on the axial scans in the thoracic spinal cord, although this was not statistically significant.

The inter-rater agreement for all three evaluated sequences was good. However, when looking at lesions detected in the sagittal T2w-TSE images plus in an additional sequence, the inter-rater agreement was higher for the sagittal T2w-TSE sequence in combination with the axial T2w-TSE sequence compared to the STIR sequence. This is somehow surprising, since former studies have reported highest inter-rater agreement for the STIR sequence [15, 17]. 

We used a 3 T scanner in our study which could explain our lower inter-rater agreement for the STIR sequence. Compared to 1.5T, 3 T MRI is more susceptible to artifacts arising from B1 field inhomogeneity, susceptibility effects, and vascular pulsations, which decreases diagnostic performance especially in the thoracic spinal cord [18, 19]. Furthermore, we only excluded patients with excessive motion artifacts but still included patients with suboptimal image quality caused by movement, vascular pulsations or adiposity. In doing so, we were able to evaluate the sequences in their clinical and routine applications.

Historically, due to time consuming acquisitions of a full coverage of the spinal cord with an axial T2w sequence, it was often limited to the cervical spinal cord [20, 21]. Recent studies that investigated long or full axial coverages of the spinal cord in MS patient have shown that lesions also occur frequently in the thoracic spinal cord and are not predominantly limited to the cervical segments [10, 12]. For example, Galler et al. who used a full axial coverage of the spinal cord reported that 47.3% of all lesions found were located below C7 which is in line with our results as we detected 46.1% of all lesion within the thoracic spinal cord. With the advantage of parallel image acquisitions, the scan time for a full axial coverage of the spinal cord was 6:46 min, which is only slightly more time consuming than the STIR sequence (5:36 min). We are confident that with recently introduced imaging techniques, such as compressed sensing or deep learning-based acceleration, acquisition times can be shortened even further while maintaining or even improving image quality.

There are some limitations which we would like to address. A significant limitation of this study, consistent with previous research comparing MR imaging sequences for lesion detection in the spinal cord, was the absence of a definitive reference standard for confirming lesion presence. A lesion size of at least 3 mm has been suggested but we also included lesions with smaller diameters [2]. Without postmortem pathological data—the only truly reliable reference—we relied on the consensus evaluations of two experienced raters which is in line with previous studies [4, 10, 12, 15, 22]. However, due to the lack of a real ground truth, we refrained from determining false positive rates, limiting conclusions about diagnostic accuracy. The study sample included a broad spectrum of MS patients and those with suspected demyelinating disease, which may limit conclusions about the diagnostic value for specific MS subtypes or disease stages but also mirrors the real-world clinical population in MS day hospitals. Further, we did not evaluate the diagnostic performance of other suggested sequences for lesion detection but focused on the most commonly applied sequences for spinal MS imaging. While other sequences have also been proposed and scientifically studied, such as sagittal PDw, sagittal PSIR or axial T2w GRE, it has been shown that these sequences are still rarely used in clinical practice [23]. This is also true for 3D sequences which offer the possibility of multiplanar reconstructions. Future research should focus on optimization of sequence parameters such as slice thickness and pixel size at 3 T field strength as well as implementation of 3D sequences to improve diagnostic accuracy in lesion detection.

Our study demonstrates the superior detection rates of the axial T2w-TSE sequence with full spinal cord coverage over the sagittal T2w-TSE and STIR sequence for spinal cord lesions in MS or suspected demyelinating disease, while inter-rater agreement was comparable between the axial T2w-TSE and the STIR sequence. Including axial T2w-TSE images with full spinal cord coverage in standard imaging protocols may enhance the evaluation of disease burden and diagnostic processes in MS.

## Data Availability

No datasets were generated or analysed during the current study.
